# Anticerebral Ischemia-Reperfusion Injury Activity of Synthesized Puerarin Derivatives

**DOI:** 10.1155/2016/9821767

**Published:** 2016-10-11

**Authors:** Yubin Ji, Pei Jiang, Xinjia Yan

**Affiliations:** ^1^Engineering Research Center of Natural Anticancer Drugs, Ministry of Education, Harbin 150076, China; ^2^Research Center on Life Sciences and Environmental Sciences, Harbin University of Commerce, Harbin 150076, China; ^3^Harbin University of Commerce, Harbin 150076, China

## Abstract

When cerebral ischemia-reperfusion injury happened in patients, multiple pathological processes occur, such as leukocyte infiltration, platelet, and complement activation, which would result in cognitive dysfunction and inflammation. Puerarin has shown protective effect on injury of neural cell. In order to enhance this protective effect of puerarin, puerarin derivatives with different log⁡*P* values were designed and synthesized. The original phenolic hydroxyl in the puerarin molecules was substituted in order to change the blood-brain barrier permeability and thus enhance the efficacy for preventing cerebral ischemia/reperfusion injury. And the structure of the newly synthesized molecules was confirmed by 1H NMR spectroscopy and mass spectrometry. The mouse model of cerebral artery ischemia/reperfusion injury was established to test the anticerebral ischemia-reperfusion injury activity of the puerarin derivatives. The assays of the water maze, Y maze, brain cortex Ca^2+^-Mg^2+^-ATP enzyme, and iNOS enzyme activity were performed in this mouse model. The results showed that puerarin derivative P1-EA and P2-EA were resulting in an increased lipophilicity that enabled the derivatives to pass more efficiently through the blood-brain barrier, thus, improving the protective effects against cerebral ischemia/reperfusion injury. Therefore, derivatives of puerarin may serve as promising approach to improve neuron function in ischemia-reperfusion brain injury-related disorders.

## 1. Introduction

Vascular dementia (VaD) is characterized by a decline in brain function resulting in a cognitive impairment syndrome that is caused by various cerebrovascular diseases such as ischemic, hemorrhagic, chronic, and polar hypoxic cerebrovascular diseases. The severe damage that VaD causes to the brain parenchyma greatly affects patients' learning ability and memory function, as well as their daily lives and health [1]. Studies have shown that VaD is frequently caused by ischemia-reperfusion injury. Global aging and the increasing incidence of VaD have resulted in increased efforts toward its prevention and treatment to improve the quality of life for aging adults. In addition, these efforts support the goals of the World Health Organization to promote “healthy aging.” Therefore, the study of VaD pathogenesis and development of effective treatment options have become an important issue in pharmaceutical and clinical research.

Puerarin is an isoflavone that has been extracted from the dried roots of the* Pueraria lobata* or* Pueraria thomsonii* Benth. legumes. It is chemically known as 8-*β*-D-glucopyranoside 4′-7-dihydroxyisoflavone (molecular formula C_21_H_20_O_9_, molecular weight of 416). Puerarin is one of the active ingredients in Chinese herbal medicine. It has a wide range of pharmacological effects and is generally used for the treatment of cardiovascular and cerebrovascular diseases. Studies have shown that puerarin reduced cerebral edema in rats with cerebral ischemia-reperfusion injury, removed lipid peroxidation products, enhanced antioxidant capacity, improved antioxidant activity of the brain tissue, and reduced the degree of focal cerebral ischemic injury [2–4]. However, the development of new drugs to treat central nervous system disorders is limited by many factors, primarily, delivery to the brain through the blood-brain barrier. The clinical use of puerarin is limited by its low solubility in water and lipids. Therefore, puerarin derivatives with different log⁡*P* values were designed and synthesized by structural modification. An ethyl acetate and acetyl amino group were linked to the phenolic hydroxyl at the 7/4′ position of the puerarin molecule to produce 7-mono- and 7,4′-disubstituted derivatives of puerarin. Four novel compounds were therefore synthesized. In addition, we tested the anticerebral ischemia-reperfusion injury activity of these puerarin derivatives in order to determine their permeability through the blood-brain barrier and pharmacological activity. The results reported here provide insight into the application and development of puerarin derivatives for the treatment of vascular dementia.

## 2. Materials and Methods

### 2.1. Experimental Animals

Male Kunming mice (SCXK (Army) 2013-004, weighing 25~30 g) maintained under controlled lighting and temperature conditions were used in this experiment. All studies on animals were performed with approval by the Institutional Animal Care and Use Committee (IACUC) of the Harbin University of Commerce.

### 2.2. Synthesis of P1-EA/P2-EA

Puerarin (15 g, 36 mmol) was dissolved in anhydrous DMF (200 mL) and then K_2_CO_3_ (29.8 g, 216.3 mmol) was added at room temperature. After stirring for 90 min, ethyl bromoacetate (35.8 g, 216 mmol) was added and the mixture was stirred at room temperature for an additional 4 h. The mixture was filtered to remove insoluble solids and then solvents were removed using a rotavapor until the mixture was in an oily state; the oily solute was then solubilized with anhydrous ether. After one more round of rotovapping ether solution, the remains were spin-dried and then dissolved in anhydrous methanol and isolated by silica gel column chromatography.

**P1-EA** (ethyl 2-[4-[7-(2-ethoxy-2-oxoethoxy)-4-oxo-8-[(2S,3R,4R,5S,6R)-3,4,5-trihydroxy-6-(hydroxymethyl)oxan-2-yl]chromen-3-yl]phenoxy]acetate): log⁡*P*-0.36; ESI-MS:* m/z* 589.2 [M + H]^+^, ^1^H-NMR (300 MHz, DMSO-d_6_) *δ*_H_8.49 (1H, s, H-2), 8.07 (1H, d, *J* = 8.7 Hz, H-5), 7.19 (1H, d, *J* = 8.7 Hz, H-6), 7.53 (1H, d, *J* = 9.0 Hz, H-2′, 6′), 6.98 (1H, d, *J* = 9.0 Hz, H-3′, 5′), 4.88 (2H, s, H-1′′), 4.22 (2H, m, H-3′′), 1.25 (3H, m, H-4′′′), 4.88 (2H, s, H-1′′), 4.22 (2H, m, H-3′′), 1.24 (3H, m, H-4′′′), 4.91 (1H, d, *J* = 9.6 Hz, H-1′′′′), 3.95 (1H, m, H-2′′′′), 3.69 (1H, m, H-3′′′′), 3.47 (1H, m, H-4′′′′), 3.30 (1H, m, H-5′′′′), 3.43 (1H, m, H-6a′′′′), 3.65 (1H, m, H-6b′′′′).** P2-EA** (ethyl 2-((3-(4-hydroxyphenyl)-4-oxo-8-((2S,3R,4R,5S,6R)-3,4,5-trihydroxy-6-(hydroxymethyl)tetrahydro-2H-pyran-2-yl)-4H-chromen-7-yl)oxy)acetate): log⁡*P*-0.23; ESI-MS:* m/z* 503.1 [M + H]^+^, ^1^H-NMR (300 MHz, DMSO-d_6_) *δ*_H_8.43 (1H, s, H-2), 8.07 (1H, d, *J* = 9.0 Hz, H-5), 7.18 (1H, d, *J* = 9.0 Hz, H-6), 7.43 (1H, d, *J* = 8.7 Hz, H-2′, 6′), 6.81 (1H, d, *J* = 8.7 Hz, H-3′, 5′), 4.88 (2H, s, H-1′′), 4.22 (2H, m, H-3′′), 1.25 (3H, m, H-4′′), 4.91 (1H, d, *J* = 9.6 Hz, H-1′′′), 3.95 (1H, m, H-2′′′), 3.69 (1H, m, H-3′′′), 3.47 (1H, m, H-4′′′), 3.30 (1H, m, H-5′′′), 3.43 (1H, m, H-6a′′′), 3.65 (1H, m, H-6b′′′), 9.55 (1H, s, -OH).

### 2.3. Synthesis of P1-acmid

P1-EA (8.82 g, 15 mmol) was added to a 200 mL sealed tube. Ammonia-containing methanol (7 mol/L, 100 mL, 700 mmol) was then added and stirred at room temperature overnight. The large amount of solids that precipitated in the solution was filtered and washed with ethanol to produce a pale yellow pellet weighing 4.5 g.

P1-acmid (2-(4-(7-(2-amino-2-oxoethoxy)-4-oxo-8-((2S,3R,4R,5S,6R)-3,4,5-trihydroxy-6-(hydroxymethyl)tetrahydro-2H-pyran-2-yl)-4H-chromen-3-yl)phenoxy)acetamide): log⁡*P*-2.73; ESI-MS:* m/z* 531.1 [M + H]^+^, ^1^H-NMR (300 MHz, DMSO-d_6_) *δ*_H_8.41 (1H, s, H-2), 8.10 (1H, d,* J* = 9.0 Hz, H-5), 7.28 (1H, d,* J* = 9.0 Hz, H-6), 7.49 (1H, d,* J* = 8.7 Hz, H-2′, 6′), 6.99 (1H, d,* J* = 8.7 Hz, H-3′, 5′), 4.50~4.77 (2H, m, H-1′′), 8.08 (1H, d,* J* = 9.0 Hz, 2′′-NH_2_), 7.16 (1H, d,* J* = 9.0 Hz, 2′′-NH_2_), 4.49~4.76 (2H, m, H-1′′′), 7.54 (1H, d,* J* = 8.4 Hz, 2′′′-NH_2_), 6.99 (1H, d,* J* = 8.4 Hz, 2′′′-NH_2_), 4.91 (1H, d,* J* = 9.6 Hz, H-1′′′′), 3.95 (1H, m, H-2′′′′), 3.69 (1H, m, H-3′′′′), 3.47 (1H, m, H-4′′′′), 3.30 (1H, m, H-5′′′′), 3.43 (1H, m, H-6a′′′′), 3.65 (1H, m, H-6a′′′′).

### 2.4. Synthesis of P2-acmid

P2-EA (7.53 g, 15 mmol) was added to a 200 mL sealed tube. Ammonia-containing methanol (7 mol/L, 100 mL, and 700 mmol) was then added and stirred at room temperature overnight. The large amount of solids that precipitated in the solution was filtered and washed with ethanol to produce a pale yellow pellet weighing 4.3 g.

P2-acmid (2-((3-(4-hydroxyphenyl)-4-oxo-8-((2S,3R,4R,5S,6R)-3,4,5-trihydroxy-6-(hydroxymethyl)tetrahydro-2H-pyran-2-yl)-4H-chromen-7-yl)oxy)acetamide): log⁡*P*-1.61; ESI-MS:* m/z *474.1 [M + H]^+^, ^1^H-NMR (300 MHz, DMSO-d_6_) *δ*_H_8.34 (1H, s, H-2), 8.09 (1H, d, *J* = 9.0 Hz, H-5), 7.24 (1H, d,* J* = 9.0 Hz, H-6), 7.36 (1H, d,* J* = 8.4 Hz, H-2′, 6′), 6.80 (1H, d,* J* = 8.4 Hz, H-3′, 5′), 4.77 (1H, d,* J* = 15.0 Hz, H-1a′′), 4.50 (1H, d,* J* = 15.0 Hz, H-1b′′), 8.07 (1H, d,* J* = 9.0 Hz, -NH_2_), 7.15 (1H, d,* J* = 9.0 Hz, -NH_2_), 4.91 (1H, d,* J* = 9.9 Hz, H-1′′′), 3.95 (1H, m, H-2′′′), 3.69 (1H, m, H-3′′′), 3.47 (1H, m, H-4′′′), 3.30 (1H, m, H-5′′′), 3.43 (1H, m, H-6a′′′), 3.65 (1H, m, H-6a′′′).

**P1-EA**: log⁡*P*-0.36; ESI-MS:* m/z* 589.2 [M + H]^+^, ^1^H-NMR (300 MHz, DMSO-d_6_) *δ*_H_8.49 (1H, s, H-2), 8.07 (1H, d, *J* = 8.7 Hz, H-5), 7.19 (1H, d, *J* = 8.7 Hz, H-6), 7.53 (1H, d, *J* = 9.0 Hz, H-2′, 6′), 6.98 (1H, d, *J* = 9.0 Hz, H-3′, 5′), 4.88 (2H, s, H-1′′), 4.22 (2H, m, H-3′′), 1.25 (3H, m, H-4′′′), 4.88 (2H, s, H-1′′), 4.22 (2H, m, H-3′′), 1.24 (3H, m, H-4′′′), 4.91 (1H, d, *J* = 9.6 Hz, H-1′′′′), 3.95 (1H, m, H-2′′′′), 3.69 (1H, m, H-3′′′′), 3.47 (1H, m, H-4′′′′), 3.30 (1H, m, H-5′′′′), 3.43 (1H, m, H-6a′′′′), 3.65 (1H, m, H-6b′′′′).** P2-EA**: log⁡*P*-0.23; ESI-MS:* m/z* 503.1 [M + H]^+^, ^1^H-NMR (300 MHz, DMSO-d_6_) *δ*_H_8.43 (1H, s, H-2), 8.07 (1H, d, *J* = 9.0 Hz, H-5), 7.18 (1H, d, *J* = 9.0 Hz, H-6), 7.43 (1H, d, *J* = 8.7 Hz, H-2′, 6′), 6.81 (1H, d, *J* = 8.7 Hz, H-3′, 5′), 4.88 (2H, s, H-1′′), 4.22 (2H, m, H-3′′), 1.25 (3H, m, H-4′′), 4.91 (1H, d, *J* = 9.6 Hz, H-1′′′), 3.95 (1H, m, H-2′′′), 3.69 (1H, m, H-3′′′), 3.47 (1H, m, H-4′′′), 3.30 (1H, m, H-5′′′), 3.43 (1H, m, H-6a′′′), 3.65 (1H, m, H-6b′′′), 9.55 (1H, s, -OH).

P1-acmid: log⁡*P*-2.73; ESI-MS:* m/z* 531.1 [M + H]^+^, ^1^H-NMR (300 MHz, DMSO-d_6_) *δ*_H_8.41 (1H, s, H-2), 8.10 (1H, d,* J* = 9.0 Hz, H-5), 7.28 (1H, d,* J* = 9.0 Hz, H-6), 7.49 (1H, d,* J* = 8.7 Hz, H-2′, 6′), 6.99 (1H, d,* J* = 8.7 Hz, H-3′, 5′), 4.50~4.77 (2H, m, H-1′′), 8.08 (1H, d,* J* = 9.0 Hz, 2′′-NH_2_), 7.16 (1H, d,* J* = 9.0 Hz, 2′′-NH_2_), 4.49~4.76 (2H, m, H-1′′′), 7.54 (1H, d,* J* = 8.4 Hz, 2′′′-NH_2_), 6.99 (1H, d,* J* = 8.4 Hz, 2′′′-NH_2_), 4.91 (1H, d,* J* = 9.6 Hz, H-1′′′′), 3.95 (1H, m, H-2′′′′), 3.69 (1H, m, H-3′′′′), 3.47 (1H, m, H-4′′′′), 3.30 (1H, m, H-5′′′′), 3.43 (1H, m, H-6a′′′′), 3.65 (1H, m, H-6a′′′′).

P2-acmid: log⁡*P*-1.61; ESI-MS:* m/z* 474.1 [M + H]^+^, ^1^H-NMR (300 MHz, DMSO-d_6_) *δ*_H_8.34 (1H, s, H-2), 8.09 (1H, d,* J* = 9.0 Hz, H-5), 7.24 (1H, d,* J* = 9.0 Hz, H-6), 7.36 (1H, d,* J* = 8.4 Hz, H-2′, 6′), 6.80 (1H, d,* J* = 8.4 Hz, H-3′, 5′), 4.77 (1H, d,* J* = 15.0 Hz, H-1a′′), 4.50 (1H, d,* J* = 15.0 Hz, H-1b′′), 8.07 (1H, d,* J* = 9.0 Hz, -NH_2_), 7.15 (1H, d,* J* = 9.0 Hz, -NH_2_), 4.91 (1H, d,* J* = 9.9 Hz, H-1′′′), 3.95 (1H, m, H-2′′′), 3.69 (1H, m, H-3′′′), 3.47 (1H, m, H-4′′′), 3.30 (1H, m, H-5′′′), 3.43 (1H, m, H-6a′′′), 3.65 (1H, m, H-6a′′′).

### 2.5. Establishment of Vascular Dementia Mouse Model by Bilateral Carotid Artery Ligation and Reperfusion

Healthy male Kunming mice were injected with atropine intraperitoneally (IP) (2 mg/kg) followed by an intraperitoneal injection of 3.5% chloral hydrate anesthesia after 5 min. An incision was made at the center of the neck and the bilateral carotid artery (not including the vagus nerve) was isolated. Blood flow in the artery was blocked with a noninvasive arterial clamp for 15 min during which convulsions, slow deep breathing, and a rapid heartbeat were observed in the mice. Meanwhile, a 1 cm cut at the tip of the tail was made to collect 0.3 mL blood. The arterial clamp was released to restore blood flow for 10 min, and then blood flow was blocked for another 15 min. Mice were sutured when regular breathing and heartbeat were observed following reperfusion. After 48 h, mice were gavaged with the respective treatments. Mice in the model group and the sham group (the animal model which has the same surgical procedure but without vascular occlusion, nor tails bloodletting) received distilled water.

### 2.6. Behavior Test

*Y Maze*. It takes 2 days for Y maze assays: the first day for training and the next day for the test [5]. Before the test, the experiment animals were adapted to the environment, with appropriate temperature and vertical light (no shadow). The mice were given medicine 1 h before the test. In the first day, the mice were put into Y maze for free exploration for 5 min without recording. In the next test day, each mouse was put into the maze and recorded for 8 min. The entrance time and duration at each arm were calculated based on the reaction rate formula made alternately.

*Water Maze*. It takes 5 day for the assay of water maze [5]. Mice were trained six times a day with recording from the beginning to the end of time. The average of each swimming time was calculated and compared among groups. The mice were given medicine 1 h before the training. Swimming time is not more than 1 min; otherwise it will cause mice death. We train the same number of mice in each group at one time. In order to reduce the activity variation caused by time difference, we train 1 mouse from each group simultaneously. In addition, for all behavioral experiments, we used the double blind test. Behavioral tests were performed 12 days after the start of administration. It has been shown that very obvious cognitive dysfunction will be observed after 15 days in ischemia-reperfusion model. Brain-related biochemical indicators of change are more significant and did not enter the recovery period around 20 days. Therefore, we sacrifice the mice for bioassays.

### 2.7. Evaluation of Biochemical Parameters

Following reperfusion injury in the mice, the brains were dissected following a quick head decapitation and placed on ice. Any surface blood was removed by filter paper. The bilateral hippocampus and cortex were isolated, weighed, and placed in a saline solution at 4°C at a weight/volume ratio of 1 : 9 for a homogenous 10% brain tissue solution. The solution was centrifuged at 3000 r/min for 15 min to obtain the supernatant. The iNOS and Ca^2+^-Mg^2+^-ATPase activities in the cortical tissue were determined with a UV-visible spectrophotometer (UV-2102C) according to the protocol.

### 2.8. Statistical Analysis

All experimental data are represented as *x*  ± SD. ANOVA was performed by the statistical software SPSS18.0 [5]. *P* < 0.05 was considered to be statistically significant.

## 3. Results and Discussion

### 3.1. Synthesis and Characterization of Puerarin Derivatives

Small molecular weight compounds can be modified to change their physical and chemical properties in order to increase lipophilicity and therefore access the brain through the blood-brain barrier. Lipophilic drug molecules with molecular weights less than 500 can pass through the blood-brain barrier by passive diffusion. Thus, small molecule drugs can be modified by the introduction of nonpolar groups or by changing hydrophilic groups to increase lipophilicity and improve permeability through the blood-brain barrier.

**Figure 1:**
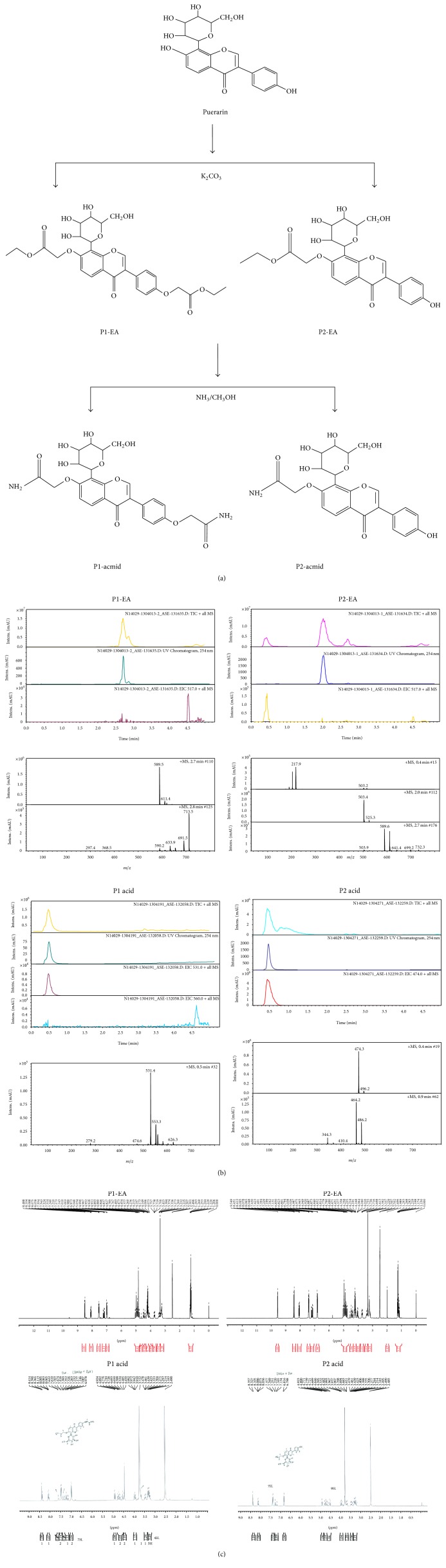
Synthesis strategy and characterization of puerarin derivatives. (a) Scheme of synthesis of puerarin derivatives. (b) MS analysis of synthetically puerarin derivatives. (c) ^1^H-NMR analysis of synthetic puerarin derivatives.

In this study, the structure of a puerarin monomer was modified by the addition of ethyl bromoacetate and then filtered to give a solid compound. The P1-EA and P2-EA products were obtained by silica gel column chromatography. P1-EA and P2-EA were then added to ammonia-containing methanol, resulting in the precipitation of a large number of light yellow solids representing P1-acmid and P2-acmid. The synthetic route is shown in Figure 1. The molecular weight and structure of the compounds were confirmed by LC-MS and ^1^H-NMR spectra, respectively. The log⁡*P* values were calculated by the fragment addition method. The results revealed that log⁡*P* values of the synthesized products, P1-EA and P2-EA, were greater than that of puerarin, indicating greater lipophilicity and better blood-brain barrier permeability.

In this study, we did not test the blood-brain barrier transparency of puerarin and its derivatives, but the octanol-water partition coefficient (i.e., log⁡*P*) of newly synthesized puerarin derivatives is calculated. Based on existing theory, structural modification to improve the physical and chemical properties of small molecule drugs will increase the lipophilicity (i.e., log⁡*P* greater the lipophilic better) of the compound, thereby increasing the passive transport of the blood-brain barrier permeability. Compared with log⁡*P* value of puerarin, its derivatives have greater log⁡*P* values, which suggest that the derivatives have better permeability.

### 3.2. Effects of Puerarin Derivatives on Improved Learning and Memory Functions Using a Mouse Model of Ischemia-Reperfusion-Induced Dementia

A mouse model for ischemia-reperfusion-induced dementia was established by bilateral carotid artery ligation and reperfusion. Mice were randomly divided into 7 groups of 10 mice each, including a sham group, model group, puerarin at 100 mg/kg group [2–4], puerarin derivative P1-EA at 100 mg/kg group, puerarin derivative P2-EA at 100 mg/kg group, puerarin derivative P1-acmid at 100 mg/kg group, and puerarin derivative P2-acmid at 100 mg/kg group. We then determined the antivascular dementia activity of puerarin and puerarin derivatives.

In addition, we measured the effect of puerarin and puerarin derivatives on learning and memory functions in ischemic reperfusion-induced dementia mice using water maze and Y maze. When bilateral carotid artery occlusion model is successfully established, the brain lesions and neurological apoptosis induced learning and memory dysfunction in mice but did not cause changes in other physical features. Some of the mice may have some dysfunction of facial nerve, such as squinting one eye. However, it did not affect the function of the body physical activity and does not affect the behavior behind the results of experiments.

**Figure 2:**
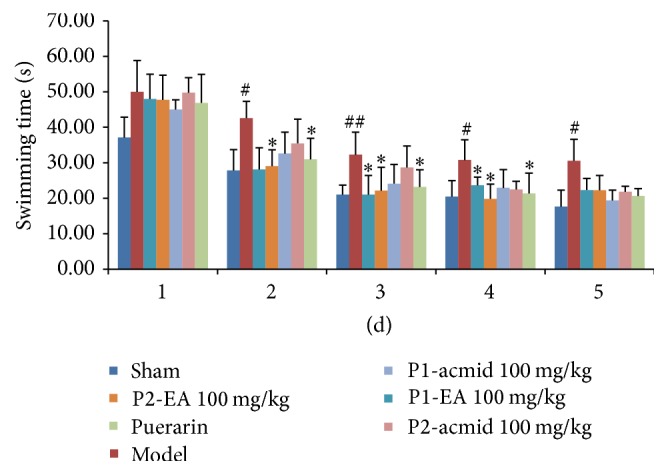
Effects of puerarin derivatives on swimming time in water maze test in mice with cerebral ischemia-reperfusion. The mice treated with different compounds performed water maze test after ischemic reperfusion injury at 1–5 days. Their swim duration time was recorded and analyzed. Each column represents the mean ± SD of 10 animals. ^##^ *P* < 0.01; ^#^ *P* < 0.05 versus sham group; ^*∗*^ *P* < 0.05 versus model group.

Water maze experiments directly reflect the spatial orientation learning and memory functions of experimental animals. The water maze results demonstrate a significant increase in the time required for the ischemic reperfusion injured mice to swim to a platform compared to the sham group, indicating that the model was established (Figure 2). The swim duration was shortened in mice given the puerarin derivatives P1-EA at 100 mg/kg and P2-EA at 100 mg/kg compared to mice in the model group. Moreover, the swim duration was significantly reduced in mice given both puerarin derivatives P1-EA at 100 mg/kg and P2-EA at 100 mg/kg on days 3 and 4 of training (*P* < 0.05). The effects observed in the puerarin derivative groups were stable and superior to the results observed in mice given puerarin only, indicating that puerarin derivatives improved learning and memory impairments after ischemia-reperfusion injury.

**Figure 3:**
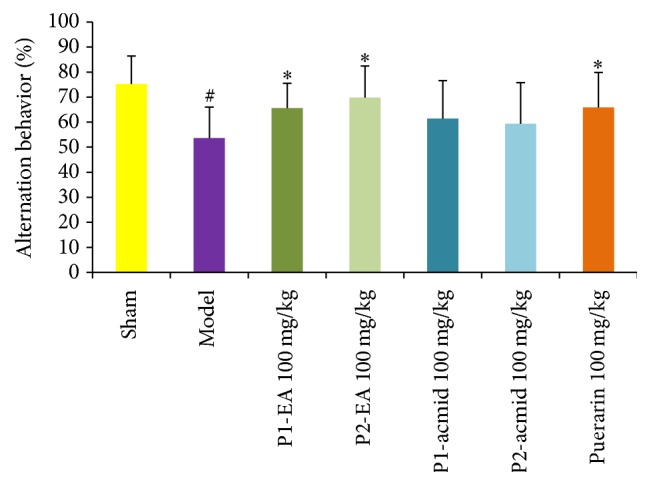
Effects of puerarin derivatives on the spontaneous alternation behavior in the Y maze test in mice with cerebral ischemia-reperfusion. The mice treated with different compounds performed Y Maze Spontaneous Alternation Test after ischemic reperfusion injury. Each column represents the mean ± SD of 10 animals. ^#^ *P* < 0.05 versus sham group; the alternation behavior of sham group is significantly different from that of model group; ^*∗*^ *P* < 0.05 versus model group.

The Y maze test is a good measurement of the spatial memory capacity of small animals as measured by the spontaneous alternation response rate that reflects the memory of space in the mice. The Y maze test results showed that the spontaneous alternation response rate in the ischemia-reperfusion injured mice was significantly reduced compared to the sham group (*P* < 0.05), which demonstrated that the model was established (Figure 3). Compared with the model group, the spontaneous alternation response rates of P1-EA at 100 mg/kg and P2-EA at 100 mg/kg groups were significantly increased, indicating that P1-EA at 100 mg/kg and P2-EA at 100 mg/kg improved learning and memory impairments caused by ischemia-reperfusion injury.

### 3.3. Effects of Puerarin Derivatives on Cortex Ca^2+^-Mg^2+^-ATPase Activity Using a Mouse Model of Ischemia-Reperfusion-Induced Dementia

ATP is required in the brain to maintain the ion balance across the membrane. In general, ATP-dependent ion pumps (Na^+^-K^+^-ATPases and Ca^2+^-Mg^2+^-ATPases) consume about 50% of the ATP in neurons. The activity of membrane enzymes is reduced when there is a shortage of energy in the form of ATP. Decreased Ca^2+^-Mg^2+^-ATPase activity results in increased ischemia-reperfusion injury due to an increase in the intracellular Ca^2+^, abnormal calcium-dependent physiological and biochemical reactions, and depletion of ATP and generation of free radicals that directly induces cell death.

**Figure 4:**
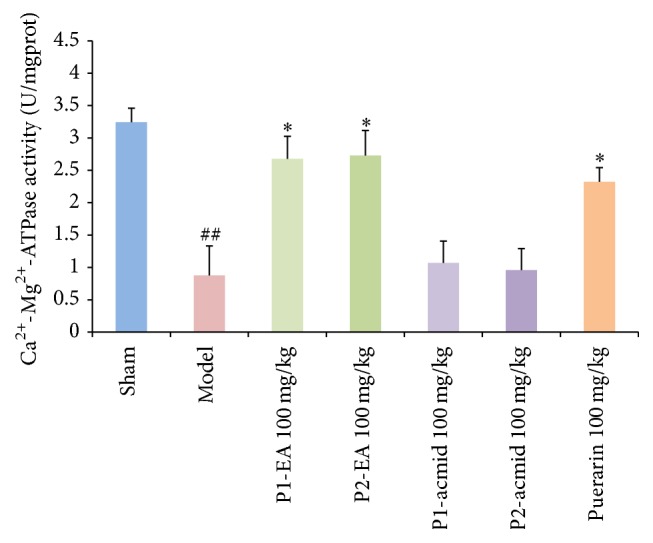
Effects of puerarin derivatives on the activity of Ca^2+^-Mg^2+^-ATPase in the cerebral cortex of mice with cerebral ischemia. Each column represents the mean ± SD of 10 animals. ^##^ *P* < 0.01 versus sham group; ^*∗*^ *P* < 0.05 versus model group.

The biochemical indicators were determined in mice following the completion of the behavioral experiments outlined in the above section. Results showed that the cerebral cortex Ca^2+^-Mg^2+^-ATPase activity in the model group decreased significantly compared to the sham group (*P* < 0.01) (Figure 4). In contrast, the Ca^2+^-Mg^2+^-ATPase activity in mice given the puerarin derivatives P1-EA at 100 mg/kg and P2-EA at 100 mg/kg was significantly higher than the model group (*P* < 0.05). These data suggest that puerarin derivatives may increase cerebral cortex Ca^2+^-Mg^2+^-ATPase activity to counter the ischemia-reperfusion injury.

### 3.4. Effects of Puerarin Derivatives on Cortex iNOS Activity in a Mouse Model of Ischemia-Reperfusion-Induced Dementia

**Figure 5:**
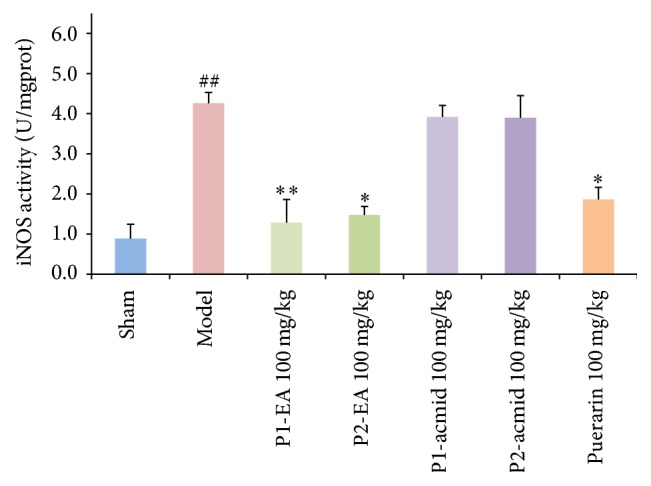
Effects of puerarin derivatives on the activity of iNOS in the cerebral cortex of mice with cerebral ischemia. Each column represents the mean ± SD of 10 animals. ^##^ *P* < 0.01 versus sham group; ^*∗*^ *P* < 0.05; ^*∗∗*^ *P* < 0.01 versus model group.

iNOS and COX-2 are two important proteins in the NF-*κ*B signaling pathway, and their mutual coordination makes them key proteins in the process of inflammation. They are highly expressed in the brain of mice with ischemia and reperfusion injury [6]. The mechanism underlying their increased expression may be related to ischemia-reperfusion-induced inflammation and oxidative stress in the brain [7, 8]. The results of our study showed that cortex iNOS activity in the model group was significantly increased compared to the sham group (Figure 5). Compared with the model group, iNOS activities in mice given the puerarin derivatives P1-EA at 100 mg/kg and P2-EA at 100 mg/kg were significantly reduced compared to the model group, suggesting that the puerarin derivatives P1-EA at 100 mg/kg and P2-EA at 100 mg/kg can reduce iNOS activity in the cerebral cortex and inhibit ischemia-reperfusion-induced inflammation. These data also suggest that the puerarin derivatives described here have anti-ischemia-reperfusion injury characteristics.

Long-term brain perfusion will lead to significant damage of learning and memory region and cause the gradual emergence of cognitive dysfunction. Therefore, cognitive dysfunction can be used as a proof for the establishment of the model. Cerebral ischemia/reperfusion injury generates series of pathological changes in the brain, for example, cerebral ischemia, ischemic infarct, uneven distribution of hippocampal CA1 neurons, and cell body shrinkage, cytoplasmic condensation, and nuclear condensation stained massive necrosis. These changes can be detected by HE staining and other immunohistochemical methods. But mice were used in this study, and mice have a relatively small brain, which is not conducive to this type of experiment, so there is no pathological test. This is a big limit of this study. In future experiments, we will establish a rat model to check the effect of puerarin derivative and perform the pathological examination after the treatment.

This study suggests that the puerarin derivatives shows enhanced neuroprotective effect of on cerebral ischemic damage in ischemia-reperfusion injury mouse. It is probably caused by the increased log⁡*P* value which suggests the derivatives are more lipophilic and, consequently, better permeabilized through blood-brain barrier. Compared with puerarin, the directives, P1-EA and P2-EA, show stronger inhibition of inflammatory responses (i.e., iNOS) and enhanced Ca^2+^-Mg^2+^-ATPase activity. Neuroprotection effect of puerarin and its derivatives is related to inflammation, NO, and Ca^2+^-Mg^2+^-ATPase. These may lead to neurobehavioral improvement in patients. Thus, treatment with puerarin derivatives may shed light on a new way of improving neuron function after ischemia-reperfusion brain injury.

## 4. Conclusion

In this study, ethyl acetate and acetyl amino groups were linked to the phenolic hydroxyl of puerarin molecules at the 7/4′ position in order to produce 7-mono- and 7,4′-disubstituted derivatives of puerarin, to generate four novel compounds. P1-EA and P2-EA showed prominent anti-ischemia-reperfusion injury effects that may be associated with the change in their log⁡*P* values. Compared with puerarin, the newly synthesized derivatives had better blood-brain barrier permeability and thus an enhanced pharmacological effect.
